# Uneven HAK/KUP/KT Protein Diversity Among Angiosperms: Species Distribution and Perspectives

**DOI:** 10.3389/fpls.2016.00127

**Published:** 2016-02-09

**Authors:** Manuel Nieves-Cordones, Reyes Ródenas, Alain Chavanieu, Rosa M. Rivero, Vicente Martinez, Isabelle Gaillard, Francisco Rubio

**Affiliations:** ^1^Biochimie et Physiologie Moléculaire des Plantes, Institut de Biologie Intégrative des Plantes, UMR 5004 CNRS/UMR 0386 INRA/Montpellier SupAgro/Université Montpellier 2Montpellier, France; ^2^Department of Plant Nutrition, Centro de Edafología y Biología Aplicada del Segura, Consejo Superior de Investigaciones CientíficasMurcia, Spain; ^3^Institut des Biomolécules Max Mousseron, UMR 5247, Faculté de PharmacieMontpellier, France

**Keywords:** HAK/KUP/KT, transporter, potassium, phylogeny, angiosperm

## Abstract

HAK/KUP/KT K^+^ transporters have been widely associated with K^+^ transport across membranes in bacteria, fungi, and plants. Indeed some members of the plant HAK/KUP/KT family contribute to root K^+^ uptake, notably at low external concentrations. Besides such role in acquisition, several studies carried out in *Arabidopsis* have shown that other members are also involved in developmental processes. With the publication of new plant genomes, a growing interest on plant species other than *Arabidopsis* has become evident. In order to understand HAK/KUP/KT diversity in these new plant genomes, we discuss the evolutionary trends of 913 HAK/KUP/KT sequences identified in 46 genomes revealing five major groups with an uneven distribution among angiosperms, notably between dicotyledonous and monocotyledonous species. This information evidenced the richness of crop genomes in HAK/KUP/KT transporters and supports their study for unraveling novel physiological roles of such transporters in plants.

## Introduction

Potassium is an essential macronutrient for plants, making up to 2–7% of the plant’s total dry weight (Evans and Sorger, 1966; Leigh and Wyn Jones, 1984). It fulfills a number of important functions, such as enzyme activation, neutralization of negative charges and, more specific to plants, the maintenance of cell turgor that leads to plant growth and organ movement (Marschner, 2012). As sessile organisms, plants need to take up K^+^ from the soil. This is firstly achieved by root epidermal and cortical cells. Then, K^+^ is loaded in the stele and transported to the shoot and distributed to the leaves (Ahmad and Maathuis, 2014; Wigoda et al., 2014). Potassium short- and long-distance transport involves the movement of K^+^ through cell membranes, notably the plasma membrane which in many cases occurs against steep concentration gradients (like in the root–soil interface, for instance). In plants, there are five major multi-gene families that encode K^+^-permeable transport systems: (i) Shaker-like K^+^ channels, (ii) tandem-pore K^+^ (TPK) channels, (iii) HAK/KUP/KT transporters, (iv) HKT transporters, and (v) cation-proton antiporters (CPAs; Mäser et al., 2001). They have become the essentials of the K^+^ transport toolkit during terrestrial plant evolution due to their widespread presence in different land plant lineages (Gomez-Porras et al., 2012).

Here we focus on the HAK/KUP/KT (High-Affinity K^+^/K^+^ UPtake/K^+^ Transporter) transporter family. Plant HAK/KUP/KT transporters were first identified in barley and *Arabidopsis* (Quintero and Blatt, 1997; Santa-María et al., 1997; Fu and Luan, 1998; Kim et al., 1998) from their homology to bacterial KUP and fungal HAK transporters (Schleyer and Bakker, 1993; Bañuelos et al., 1995). Due to the different acronyms used in these early reports, the composite name of HAK/KUP/KT is widely used to refer to the whole family in plants. Plant HAK/KUP/KT proteins possess 10–15 transmembrane (TM) segments with both N- and C-termini in the intracellular side of the membrane, the latter being much longer (Rubio et al., 2000; Gomez-Porras et al., 2012). They have been widely shown to mediate K^+^ fluxes when expressed in K^+^-uptake deficient bacteria or yeast. Moreover, plant HAK/KUP/KT proteins differ in their affinity for K^+^ and can mediate cation influx as well as eﬄux (Fu and Luan, 1998; Rubio et al., 2000; Senn et al., 2001; Bañuelos et al., 2002; Garciadeblas et al., 2002; Ahn et al., 2004). Different studies reported that HAK/KUP/KT transporters poorly discriminate between K^+^, Rb^+^, and Cs^+^ and are inhibited by NH_4_^+^ (Santa-María et al., 1997; Rubio et al., 2000; Bañuelos et al., 2002; Martínez-Cordero et al., 2004). Plant HAK/KUP/KT proteins exhibit a great diversity in terms of subcellular localization (plasma membrane, tonoplast, or other endomembranes; Bañuelos et al., 2002; Jaquinod et al., 2007; Qi et al., 2008; Osakabe et al., 2013; Rigas et al., 2013) and expression patterns (root meristems, vascular tissues, guard cells, fruits, or specialized organs such as flytraps; Elumalai et al., 2002; Ahn et al., 2004; Vicente-Agullo et al., 2004; Davies et al., 2006; Osakabe et al., 2013; Scherzer et al., 2015).

Regarding their functions, some members of the plant HAK/KUP/KT family contribute to root K^+^ uptake, notably at low external concentrations (high-affinity range) through active K^+^ transport (Nieves-Cordones et al., 2014). Such high-affinity K^+^ transporters are expected to be H^+^/K^+^ symporters (Rodriguez-Navarro et al., 1986; Maathuis and Sanders, 1994), but experimental support for this notion is still required. Several studies carried out in *Arabidopsis* have shown that other members are involved in the regulation of cell size, auxin distribution or osmotic stress adaptation (Very et al., 2014). Such three roles highlight the great importance and role diversity of HAK/KUP/KT transporters in plant physiology besides K^+^ acquisition.

During the last two decades, research on *Arabidopsis* has notably accelerated the acquisition of information concerning the molecular and physiological mechanisms around K^+^ transport and HAK/KUP/KT proteins. This has been possible mainly because of the availability of its genome sequence and the use of T-DNA insertion lines to knock-out gene function. In recent years, genome sequences from many plant species have become available. This, together with the establishment of genome-editing techniques, such as Transcription Activator-Like Effector Nucleases (TALEN) or Clustered Regularly Interspaced Short Palindromic Repeats-Cas system (CRISPR-Cas) opens the door to investigate HAK/KUP/KT gene function in crops much faster (Andersen et al., 2015). It is true that research on crop species can benefit from the information gained in *Arabidopsis*, but the study of certain physiological processes, such as the development of a fleshy fruit, need to be carried out in appropriate species. In order to orientate further research in HAK/KUP/KT function in crop species, we present a multi-species phylogenetic analysis of plant HAK/KUP/KT proteins (comprising 913 members from 46 sequenced genomes) evidencing the presence of five major clades and remarkable specificities depending on the angiosperm group considered.

## HAK/KUP/KT Phylogeny in Angiosperms

**FIGURE 1:**
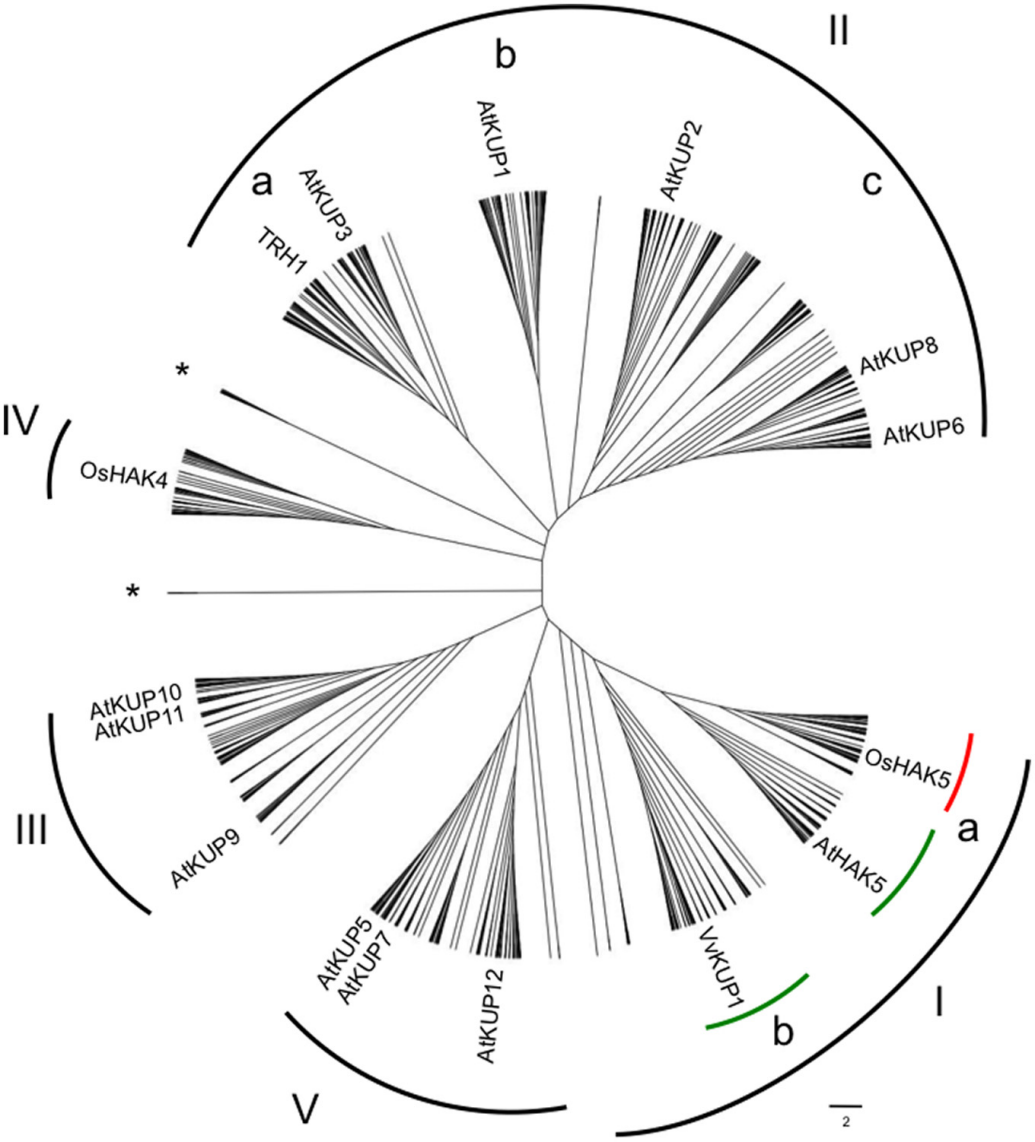
**Phylogenetic tree of the HAK/KUP/KT family in plants containing 913 sequences from 46 fully sequenced angiosperm genomes plus three outgroups (*Picea abies*, *Selaginella moellendorffii*, and *Physcomitrella patens*)**. Protein sequences fall into five main clades (I to V) where V is a novel clade. Some sub-clades within clade I contain only sequences from dicotyledonous (green lines) or monocotyledonous (red line) species. Asterisks represent outgroup sequences, which did not fall into main clades. Letters depict sub-clades within clades I and II. Representative members within the different clades are shown. Retrieved sequences from public genomic resources were ascribed to the HAK/KUP/KT family by using Orthomcl (http://orthomcl.org/orthomcl/). Protein organization was evaluated with MEME suite website (http://meme-suite.org/). Then, sequences were aligned by MAFFT (http://mafft.cbrc.jp/alignment/server/) and then alignment curation by G-block analysis in Seaview was applied prior to tree building. Tree building was constructed with MEGA6 by maximum-likelihood analysis. The scale bar represents number of substitutions per site. See also Supplementary Table S1.

Phylogenetic relationships within the HAK/KUP/KT family have consistently shown the existence of several clades in angiosperm species, but with weak biological support for such distribution (Rubio et al., 2000; Gupta et al., 2008; Gomez-Porras et al., 2012; Very et al., 2014). Since the number of sequenced angiosperm genomes, and thus that of HAK/KUP/KT available sequences, has notably increased in the last years, we wanted to assess the robustness and the species distribution of the different clades. For that purpose, we made an inventory of HAK/KUP/KT protein sequences from 43 angiosperm genomes plus three outgroup species (one gymnosperm, *Picea abies*, and two primitive non-seed plants *Selaginella moellendorffii* and *Physcomitrella patens*; Supplementary Table S1). The phylogenetic tree obtained by maximum likelihood for such sequences revealed five major clades (I to V) where I to IV followed previous numeration (Rubio et al., 2000; Gupta et al., 2008; Gomez-Porras et al., 2012; Very et al., 2014) (**Figure 1**). Representative HAK/KUP/KT transporters that have been functionally characterized are found throughout the tree. Several subgroups were identified in clade I (Ia and Ib) and in clade II (IIa, IIb, and IIc). Then, we assessed the HAK/KUP/KT sequence distribution in the different analyzed species and the angiosperm orders to which they belong (**Table 1**). Results from the common ancestor of dicotyledonous and monocotyledonous species, *Amborella trichopoda*, evidenced the presence of HAK/KUP/KT transporters in that ancestor in all of the aforementioned clades. They also suggested that clade I separation into Ia and Ib occurred at the beginning of the angiosperm lineage since *A. trichopoda* has Ia and Ib transporters and outgroup sequences belonging to clade I were not placed in any of this two major subclades. It is worth to note that clade Ib only contained sequences from dicotyledonous species, but not from monocotyledonous ones (**Figure 1**, **Table 1**). This result suggests that clade Ib disappeared in the monocotyledonous lineage because, as stated before, it was already present in the *A. trichopoda* genome. Within HAK/KUP/KT transporters from dicotyledonous genomes, different transporter distributions among orders were identified and, in some cases, groups of related species displayed empty clades that are indicative of important events in the evolution of HAK/KUP/KT transporters in dicotyledonous orders. Indeed, in Solanales, clade IIb transporters were not identified, while in Cucurbitales it was the case for clade IV. The analysis of HAK/KUP/KT transporters from Brassicales species provided striking results: clades Ib and IV were absent in HAK/KUP/KT transporters from the Brassicaceae family (eight genomes, including *Arabidopsis thaliana*) while in *Carica papaya* (belonging to Brassicales but not to such family) had one transporter belonging to clade Ib and three in clade IV. Thus, a loss of both clades could have taken place during the evolution of Brassicaceae. At the outgroup level, HAK/KUP/KT transporters from *Physcomitrella patens* were found in clade I, IV, and V whereas 13 sequences from this organism fell apart in two separate branches independent from the five major clades. With respect to *Selaginella moellendorffii* and *Picea abies*, we did not observe sequences in clades IIa and IIb in the former and in clade III in the latter.

**Table 1:** HAK/KUP/KT gene distribution among angiosperm orders and outrgroups.

Group	Order	Species	Clade								
			Ia	Ib	IIa	IIb	IIc	III	IV	V	Total
	Amborellales	*Amborella trichopoda*	1	2	2	1	4	1	2	2	15
Dicotyledons	Solanales	*Solanum lycopersicum*	2	3	3	0	5	2	2	4	21
		*Solanum tuberosum*	2	2	3	0	4	2	0	2	15
	Lamiales	*Mimulus guttatus*	0	3	2	0	4	1	1	2	13
	Vitales	*Vitis vinifera*	1	4	2	2	5	1	1	2	18
	Fabales	*Cicer arietinum*	1	1	2	2	4	1	1	3	15
		*Glycine max*	3	1	6	3	10	3	1	5	32
		*Medicago truncatula*	3	2	2	3	4	3	0	3	20
		*Phaseolus vulgaris*	2	0	3	1	6	2	1	3	18
	Rosales	*Fragaria vesca*	3	1	2	2	4	2	3	0	17
		*Malus domestica*	3	6	0	3	7	3	2	4	28
		*Prunus persica*	2	1	1	2	4	3	0	2	15
	Cucurbitales	*Cucumis melo*	1	4	1	1	4	2	0	2	15
		*Cucumis sativus*	1	4	1	1	4	2	0	2	15
	Malpighiales	*Jatropha curcas*	1	2	2	1	3	1	1	1	12
		*Linum usitatissimum*	1	2	2	1	9	5	0	3	23
		*Manihot esculenta*	1	5	3	1	7	1	1	2	21
		*Populus trichocarpa*	3	1	3	4	8	4	2	4	29
		*Ricinus communis*	1	2	2	1	4	1	1	2	14
		*Salix purpurea*	3	1	3	2	6	4	1	3	23
	Myrtales	*Eucalyptus grandis*	6	9	2	1	6	3	1	2	30
	Sapindales	*Citrus clementina*	1	5	2	1	3	2	1	2	17
		*Citrus sinensis*	1	3	2	2	3	2	1	2	16
	Malvales	*Gossypium raimondii*	2	3	3	3	7	2	1	5	26
		*Theobroma cacao*	1	3	2	3	4	1	1	2	17
	Brassicales	*Arabidopsis halleri*	2	0	2	1	3	3	0	3	14
		*Arabidopsis lyrata*	3	0	2	1	3	3	0	3	15
		*Arabidopsis thaliana*	1	0	2	1	3	3	0	3	13
		*Boechera stricta*	1	0	2	1	3	3	0	3	13
		*Brassica rapa*	1	0	3	2	4	6	0	3	19
		*Capsella grandis*	1	0	2	1	3	3	0	3	13
		*Capsella rubella*	1	0	2	1	4	3	0	3	14
		*Eutrema salsugineum*	1	0	2	1	3	4	0	3	14
		*Carica papaya*	0	1	1	2	4	2	3	1	14
	Ranunculales	*Aquilegia coerulea*	3	0	2	2	3	1	4	2	17
Monocotyledons	Zingiberales	*Musa acuminata*	0^∗^	0	4	3	7	5	1	3	24
	Poales	*Brachypodium distachyon*	6	0	3	1	7	3	5	2	27
		*Hordeum vulgare*	5	0	2	0	4	2	0	2	15
		*Oryza sativa*	8	0	3	1	5	3	4	3	27
		*Panicum virgatum*	21	0	6	2	12	6	4	6	57
		*Setaria italica*	12	0	3	1	5	3	3	3	30
		*Sorghum bicolor*	11	0	4	1	5	3	2	4	30
		*Zea mays*	9	0	3	1	5	3	3	3	27

**Outgroups**		**Species**	**Cluster**							
			**I**	**IIa**	**IIb**	**IIc**	**III**	**IV**	**V**	**Total**	

Gymnosperms		*Picea abies*	5	1	1	3	0	1	2	13	
Lycopodiophytes		*Selaginella moellendorffii*	2	0	0	2	4	2	1	11	
Bryophytes		*Physcomitrella patens*	1	0	0	0	0	2	2	18^∗∗^	

*Clades where no sequence was found in a given species are shown in red. ^∗^There is one protein belonging to cluster I but it is located in a different branch from Ia and Ib. ^∗∗^Thirteen sequences fell apart from the five major clades described for angiosperms*.

Despite the number of HAK/KUP/KT transporters whose physiological role has been established is relatively small, some conclusions can be drawn from the present analysis. Transporters involved in root high-affinity K^+^ uptake both from dicotyledonous or monocotyledonous species fall into clade Ia: HvHAK1, AtHAK5, OsHAK1, CaHAK1, SlHAK5/LeHAK5, and EsHAK5/ThHAK5, for instance (Santa-María et al., 1997; Bañuelos et al., 2002; Martínez-Cordero et al., 2004; Nieves-Cordones et al., 2007; Rubio et al., 2008; Aleman et al., 2009). However, recent work on rice OsHAK5 and OsHAK21, which also belong to clade Ia, showed more specialized functions when compared to the typical high-affinity K^+^ transporter OsHAK1 (Chen et al., 2015). For instance, OsHAK5 and OsHAK21 were involved in K^+^ transport to aerial parts during K^+^ deficiency or salt stress, respectively (Yang et al., 2014; Shen et al., 2015). Since rice and other grasses belonging to the Poaceae family exhibited a higher number of clade Ia HAK/KUP/KT sequences than dicotyledonous genomes (10.29 vs. 1.84), it could be interpreted as a specific diversification of Ia high-affinity K^+^ transporters in Poaceae species. It is worth to note that disruption of the *OsHAK1* gene led to a dramatic decrease in grain yield (Chen et al., 2015), whereas such a phenotype has not been observed in the *AtHAK5* KO mutant (Nieves-Cordones and Rubio, unpublished results). It would be interesting to know which is the contribution to grain yield of OsHAK1-like transporters from other cereal species, since they could be good targets to improve food production. Regarding clade Ib HAK/KUP/KT transporters, two reports have provided us with some information about this group. First, VvKUP1/VvHAK1-a from grapevine was shown to be expressed in flowers and grape berry skin, showing its highest expression level in the latter tissue during the pre-veraison stage (Davies et al., 2006). Second, DmHAK5 from *Dionaea muscipula* (Venus flytrap) contributes to high-affinity K^+^ uptake in digesting traps (Scherzer et al., 2015). Further characterization of clade Ib transporters will clarify whether they are specialized in transporting K^+^ in tissues other than roots. Interestingly, recent reports showed that some clade I HAK/KUP/KT transporters, including DmHAK5 (clade Ib), SlHAK5, CaHAK1, and AtHAK5 (clade Ia), are activated by CIPK23-CBL1/9 complexes, which provide novel insights into the regulation of high-affinity K^+^ transport (Ragel et al., 2015; Scherzer et al., 2015). Moreover, such regulatory network offers a new alternative that could be used to enhance K^+^ uptake in tomato and pepper plants.

Clade II has been associated in *Arabidopsis* with developmental processes, especially those which demand turgor-driven cell expansion. In clade IIa, there is AtKUP4/TRH1 (Tiny Root Hairs 1) which contributes to the polar localization of auxin transporters in the root apex that, in turn, establishes auxin gradients necessary for both gravitropic responses and root hair formation (Rigas et al., 2001, 2013; Vicente-Agullo et al., 2004). The first cloned HAK/KUP/KT transporter from *Arabidopsis*, AtKUP1/KT1, belongs to clade IIb, but no physiological role has been attributed to it so far (Quintero and Blatt, 1997; Fu and Luan, 1998; Kim et al., 1998). In clade IIc, there are AtKUP2/6/8 which have been shown to negatively regulate plant growth and cell size by mediating K^+^ eﬄux rather influx (Osakabe et al., 2013). Analysis of an AtKUP2/6/8 triple null mutant also evidenced impaired ABA responses in guard cells and lateral root cells. Phosphorylation of AtKUP6 by OST1 connected osmotic stress adaptation to the regulation of K^+^ fluxes mediated by HAK/KUP/KT transporters.

With respect the other clades, GhKT1 from cotton (clade III; *Gossypium hirsutum*) was specifically upregulated during cotton fiber elongation (Ruan et al., 2001). Regarding clade IV transporters, only two have been characterized so far. LjKUP from *Lotus japonicus* was highly expressed during late nodulation development and complemented K^+^ uptake deficient bacteria (Desbrosses et al., 2004). On the other hand, PpHAK13 from the outgroup species *Physcomitrella patens* is a high-affinity Na^+^ transporter, with low K^+^ permeability, that was repressed under the presence of high Na^+^ concentrations (Benito et al., 2012). The latter transporter raises the question whether other plant HAK/KUP/KT transporters are permeable to Na^+^ at low external concentrations. Finally, belonging to clade V, PpHAK1 from *Physcomitrella patens* was shown to regulate steady K^+^ content and plant morphology under non-K^+^-limiting conditions and to contribute to high-affinity Rb^+^ and Cs^+^ uptake during K^+^ starvation (Garciadeblas et al., 2007).

Besides their physiological roles, subcellular localization of HAK/KUP/KT transporters has been assessed in some cases and it was shown to be quite diverse. Furthermore, there is not a clear relationship between phylogenetic clade to which a transporter belongs and its targeted cell membrane. For instance, several members are targeted to the plasma membrane, such as AtHAK5, OsHAK1, OsHAK21, OsHAK5 (clade I; Qi et al., 2008; Yang et al., 2014; Chen et al., 2015; Shen et al., 2015), AtKUP6 (clade IIc; Osakabe et al., 2013), and LjKUP (clade IV; Desbrosses et al., 2004) while others are targeted to the tonoplast (OsHAK10, clade IIc, and AtKUP5 clade V; Jaquinod et al., 2007; Bañuelos et al., 2002) or endoplasmatic reticulum-like endomembranes (AtKUP4/TRH1; Rigas et al., 2013).

## Conclusion and Perspectives

Plant HAK/KUP/KT K^+^ transporters have been shown to play key roles in plant physiology like K^+^ acquisition, abiotic stress adaptation and developmental processes. Interestingly, the fact that HAK/KUP/KT transporters are permeable to K^+^ only explains a part of the phenotypes exhibited by the plants lacking them as it is the case of AtHAK5 or AtKUP4/TRH1, where energization of AtHAK5-mediated K^+^ uptake or the relationship between auxin distribution and AtKUP4/TRH1 activity deserve further attention. From our analysis, it can be deduced that the contribution of HAK/KUP/KT K^+^ transporters to plant physiology may substantially differ among species, especially when entire clades are missing in a given group of species as shown. Therefore, *Arabidopsis* can still be a good model for certain well conserved roles of HAK/KUP/KT K^+^ transporters, AtHAK5 for example, but research on other species, notably crops, is required: (i) to study transporters belonging to clades Ib or IV (missing in the Brassicaceae family) or clades where significant gene duplication occurred (clade Ia in monocots) and (ii) to investigate physiological aspects which are absent in *Arabidopsis* (fleshy fruit development, for instance). Besides, some HAK/KUP/KT proteins can be already regarded as interesting candidates for future crop improvement strategies, for example GhKT1 (specifically upregulated during cotton fiber elongation), VvKUP1/VvHAK1-a (highly expressed during the preveraison stage) and OsHAK1 (critical for rice grain yield). In line with this statement, the peach fruit is the organ where more HAK/KUP/KT genes are expressed in this species (Song et al., 2015). Further research on this transporter family will contribute to understanding how we can engineer plants for food and renewable biomass production.

## Author Contributions

MN-C, RR, and FR performed the experimental analyses. MN-C wrote the article with inputs from AC, RMR, VM, IG, and FR.

## Conflict of Interest Statement

The authors declare that the research was conducted in the absence of any commercial or financial relationships that could be construed as a potential conflict of interest.

## Supplementary material

TABLE S1 **| List of HAK/KUP/KT sequences used in **Figure 1** (Excel file)**.
